# A systematic review of association studies of common variants associated with idiopathic congenital talipes equinovarus (ICTEV) in humans in the past 30 years

**DOI:** 10.1186/s40064-016-2353-8

**Published:** 2016-06-27

**Authors:** Bi-Cheng Yong, Fu-Xing Xun, Lan-Juan Zhao, Hong-Wen Deng, Hong-Wen Xu

**Affiliations:** Department of Pediatric Orthopedics, Guang Zhou Women and Children’s Medical Center, Sun Yat-Sen University, 9th Floor, No 9., Jingsui Road, Guangzhou, China; Department of Biostatistics and Bioinformatics, School of Public Health and Tropical Medicine, Tulane University, Orleans, LA USA

**Keywords:** ICTEV, Etiology, Genetics

## Abstract

The genetic cause of idiopathic congenital talipes equinovarus (ICTEV) is largely unknown. We performed a systematic review to describe the findings from 21 studies that have examined the genetic variants related to ICTEV, and to evaluate the quality of reporting. We found that ICTEV was positively associated with Hox family genes, collagen family genes, GLI3, N-acetylation genes, T-box family genes, apoptotic pathway genes, and muscle contractile family genes. Negative and controversial results were also discussed, and several genes associated with ICTEV were identified. Due to the limitation of the included studies, rare coding variants should be further investigated, sample size should be enlarged, and candidate genes should be replicated in larger ICTEV populations. Epigenetic study, pathways, chromosome capture, and detailed gene-environment interaction will also allow further elucidation of factors involved in ICTEV pathogenesis and may shed light on diagnosis and timely and accurate interventions.

## Background

Idiopathic congenital talipes equinovarus (ICTEV), also called clubfoot, is a common orthopedic birth defect found in 1 of 1000 infants (Wynne-Davies 1964). Males are more commonly affected than females by a ratio of 2 to 1 and the incidence of bilaterality is 50 %. The highest prevalence is found in Hawaiians and Maoris, and the lowest in Chinese (Chapman et al. 2000; Chung et al. 1969). The etiology of ICTEV is largely unknown, but it is universally acknowledged that gene-environment interaction plays a major role (Lochmiller et al. 1998). A genetic component to the etiology of clubfoot has been established in several studies (Bacino and Hecht 2014). In this paper, we systematically review and summarize studies performed on ICTEV probands and families regarding susceptible genes, pathways, and epigenetic changes. Major findings in humans in the last 30 years are presented and ideas for further study are discussed.

## Methods

### Literature search strategy

This systematic review was conducted according to the guidelines of the Preferred Reporting Items for Systematic Reviews and Meta-Analyses (Moher et al. 2009). The process began with the first author (YBC) performing a systematic electronic literature search of PubMed, Web of Science, China Wanfang Med Online, and Cochrane Library, for publications from Jan 1985 to Dec 2014. Queries to identify potentially relevant publications on the genetic study of patients with ICTEV were based on Boolean combinations of the following search terms: ((Talipes Equinovarus (MeSH) OR Club Foot (MeSH)) AND (Gene (MeSH) OR Genetics (MeSH)).

### Study eligibility criteria

We limited this review to publications that were in English and Chinese, with full text available, concerning patients diagnosed with ICTEV. Family based, discordant sib pair association and case–control studies were included. Studies were excluded if they were case reports, dissertations, editorials, commentaries, or review articles.

### Data extraction

**Fig. 1:**
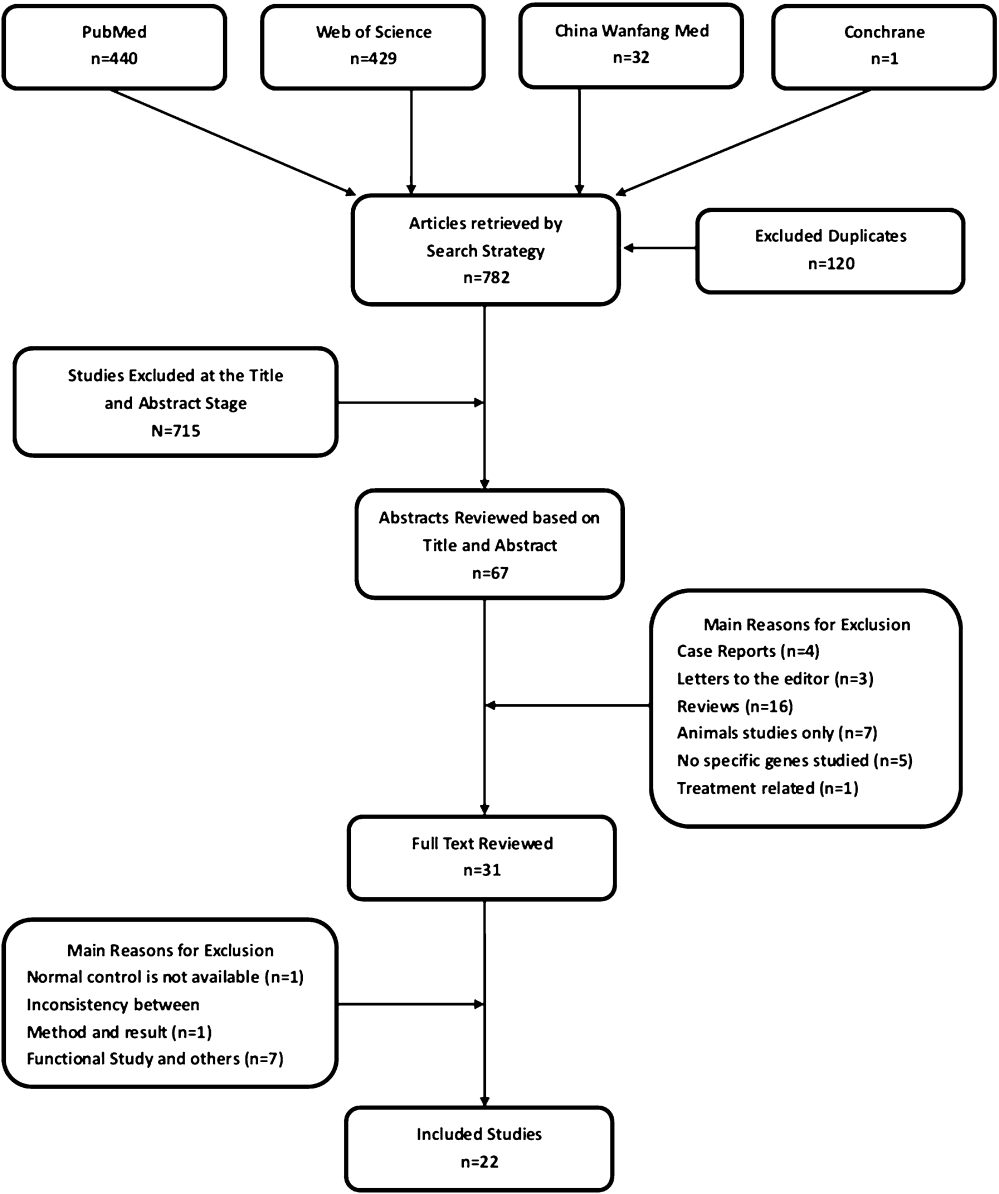
Flow diagram of the study identification and selection process

The first author (YBC) screened the titles and abstracts of all the retrieved articles to determine whether they met the eligibility criteria, and appraised the methodological quality and evidence of each selected study. The second author (XFX) subsequently reviewed the accuracy and quality of the appraisal. Any disagreements were discussed between the first and the second authors until consensus was reached. The flow diagram describes the process used to select articles for this study, and the results of the literature search (Fig. 1). The following information was extracted from each study: (1) Year of publication, (2) Study objectives, (3) Study’s inclusion and exclusion criteria, (4) Sample size, (5) Method, (6) Gene examined, (7) Association or non-association study, (8) Results, and (9) Corresponding author. Due to the heterogeneity in the study design, in this review, meta-analysis was not performed for those observational gene(s) identified among the various reviewed studies. Two investigators independently assessed the quality of the reporting. Differences in the assessment were resolved by discussion.

## Results

### Literature search

We identified 902 relevant studies from various resources (Fig. 1); the included studies examined the genetic associations between relevant genes and ICTEV. After reading the titles, 715 irrelevant articles were excluded. A total of 67 abstracts addressing certain gene(s) associated with ICTEV were selected to be reviewed. After reviewing, 36 articles did not meet our criteria and were excluded, including case reports (n = 4), letters to the editor (n = 3), reviews (n = 16), animal only studies (n = 7), no specific genes studied (n = 5), and treatment related (n = 1). For the remaining 30 articles, full texts were screened. Nine more articles were excluded due to normal control being unavailable (n = 1), inconsistency between method and result (n = 1), and functional study and other reasons (n = 7). Twenty-one studies met the predetermined inclusion criteria (Fig. 1).

### Description of the included studies (Table 1)

From the studies gathered, we identified 21 that investigated genes or pathways which may contribute to the occurrence of ICTEV. Among them, some genes are positively associated with ICTEV, while others have shown no evidence of association. The genes with positive results include: (1) Hox family genes (HoxA and HoxD), (2) collagen family genes (COL9A1 and COL1A1), (3) GLI3, (4) N-acetylation genes (NAT2), (5) T-box family genes (TBX3 and TBX4), (6) apoptotic pathway genes (Casp3, Casp8, Casp9, Casp10, Bid, Bcl-2, and Apaf1), and (7) muscle contractile family genes (TNNC2 and TPM1). The genes with negative or controversial results are CAND2, WNT7a, MYH, and DTDST. Our critical review of these genes affecting ICTEV is summarized as follows:

**Table 1:** The reporting quality of the reviewed 21 studies

Year	Study object	Study type	Inclusion criteria	Exclusion criteria	Race/Ethnicity	Sample size	Method	Gene examined	Association study	Results	Corresponding author
2002	To evaluate the relationship between R279 W mutation in DTDST and occurrence of ICTEV	Family based	ICTEV patients and their families	Associate with other anomalies and syndromes	Hispanic and Nonhispanic White	125 ICTEV probands and their parents	PCR, Genotyping	DTDST	N	Negative	Jacqueline T. Hecht
2003	To investigate possible association between ICTEV and HoxD gene	Family based	ICTEV patients and their families	Homozygous pedigree; Incomplete information	Chinese	42 ICTEV probands and their parents	PCR, Genotyping, TDT	HoxD	Y	Positive	Shi-Jun Ji
2004	To investigate correlation between ICTEV and PAX5, PAX6 and TBX3	Family based	ICTEV patients and their families	NOS	Chinese	123 ICTEV probands in 41 nuclear family trios	PCR, Genotyping, TDT	PAX5 PAX6 TBX3	Y	TBX3 Positive	Hong-wei Ma
2005	To study 2q31-33 SNP in ICTEV patients	Family based	ICTEV patients and their families	TEV from other causes	Hispanic and Nonhispanic White	57 multiplex families and 83 simplex families	PCR, Genotyping	CASP8 CASP10 CFLAR	Y	CASP10 Positive	Jacqueline T. Hecht
2005	To study SNPs in HoxD10, HoxD12, HoxD13 and haplotypes distribution in ICTEV pedigree	Family based	ICTEV patients and their families	NOS	Chinese	125 ICTEV probands	PCR, Genotyping, TDT	HoxD10 HoxD12 HoxD13	Y	HoxD12 HoxD13 Positive	Chun-lian Jin
2006	To explore the association and mutation of GLI3 gene in ICTEV	Family based	ICTEV patients and their families	NOS	Chinese	271 ICTEV probands and their parents 100 normal controls	PCR, Genotyping, TDT	GLI3	Y	GLI3 Positive	Chun-lian Jin
2006	To study MTHFR C677Tpolymorphism, and maternal periconceptional folic acid supplement use, influenced risk of isolated clubfoot	Family based	ICTEV patients and their families	syndromic TEV	Not specified	375 case-parent triads	PCR, Genotyping	MTHFR	N	MTHFR Positive	Linda Sharp
2007	To analyze SNPs within COL9A1 gene in ICTEV	Family based	ICTEV patients and their families	NOS	Chinese	252 ICTEV probands in 41 nuclear family trios	PCR, Genotyping, ETDT	COL9A1	Y	COL9A1 Positive	Chun-lian Jin
2007	To test the possible association between NAT2, NAT3 and ICTEV	Family based	ICTEV patients and their families	NOS	Hispanic and Caucasian	56 extended multiplex families, 57 trios, and 157 Hispanic and 80 white non-Hispanic simplex trios	PCR, Genotyping, PDT, FBAT	NAT2 NAT3	Y	NAT2 Positive	Jacqueline T. Hecht
2007	To study the association between Apoptotic genes and ICTEV	Family based	ICTEV patients and their families	NOS	Hispanic and Caucasian	170 Caucasian families, 179 Hispanic families	PCR, Genotyping, FBAT, PDT	Casp3 Casp8 Casp9 Casp10 Bid Bcl-2 Apaf1	Y	Tested genes positive	Jacqueline T. Hecht
2008	To study possible association between ICTEV and HoxD	Family based	ICTEV patients and their famliies	Incomplete chart records	Chinese	65 ICTEV patients 96 members from 32 families	PCR, Genotyping, TDT	HoxA	Y	HoxA Positive	Chun-lian Jin
2008	To detect the expressions of COL1A1 mRNA in 20 patients with ICTEV	Case–control	ICTEV patients	NOS	Chinese	84 ICTEV probands and their parents 100 normal controls	PCR-DGGE, DNA sequencing	COL1A1	N	COL1A1 Positive	Chun-lian Jin
2009	To test the hypothesis that CAND2 and WNT7a mutation associated with ICTEV	Case–control	ICTEV patients	Other syndromes including TEV	Not specified	256 ICTEV patients and their parents 75 matched controls	PCR, DNA sequencing	CAND2 WNT7a	N	Negative	Jose A. Morcuende
2009	To detect the association between DTDST and ICTEV	Case–control	ICTEV patients	Associate with other abnomalies and syndromes	Chinese	40 ICTEV patients 10 matched controls	RT-PCR, PCR-SSCP	DTDST	N	Positive	WU Xin-le
2009	To evaluate the expression level of CD-RAP	Case–control	ICTEV patients	Neuromuscular or syndomic TEV	Chinese	25 ICTEV patients 5 controls	RT-PCR	CD-RAP	N	Positive	Chun-lian Jin
2009	To study HoxA, HoxD and IGFBP3 in patients with ICTEV	Family based	ICTEV patients	Chromosomal abnormality or syndrome	Hispanic and Non-Hispanic White	179 extended families 331 simplex families 88 trios 144 families for validation	PCR, Genotyping, In Silico	HoxA HoxD IGFBP3	Y	Tested genes positve Interactions with CASP3	Jacqueline T. Hecht
2010	To study MYH genes in ICTEV patients	Case–control	ICTEV patients	Neuromuscular or other syndrome with TEV	Not Specified	200 patients 200 controls	PCR, DNA sequencing	MYH 1 MYH 2 MYH 3 MYH 8	N	MYH genes not directly cause ICTEV	Jose A. Morcuende
2012	To assess whether variation in or around TBX4 is a common cause of nonsyndromic clubfoot.	Family based	ICTEV patients and their families	syndromic causes of clubfoot	Hispanic and Non-Hispanic White	605 families	aCGH, PCR, Genotyping, DNA sequencing	TBX4	Y	TBX4 variation is not a frequent cause	Jacqueline T. Hecht
2012	To interrogate muscle contractile complex genes in ICTEV	Family based and case–control	ICTEV patients and their families	NOS	Hispanic and Non-Hispanic White	224 multiplex families 357 simplex families	PCR, Genotyping, DNA sequencing	Muscle contractile complex genes	Y	TNNC2 was identified in a validation group	Jacqueline T. Hecht
2014	To identify genetic risk factors associated with clubfoot	Case–control	ICTEV patients	Additional birth defects, known genetic, syndromes, developmental delay, mental retardation	Hispanic and non-Hispanic White	396 ICTEV patients 1000 controls	Microarray genotyping, GWAS association study	Genome	Y	SNPs replication 12q24.31 FOXN3, SORCS1 MMP7/TMEM123	Christina A Gurnett

### Positive gene results

#### Hox family genes

The HOX genes encode a highly conserved family of transcription factors that play fundamental roles in morphogenesis during embryonic development. This group of genes determines the segment identity and also helps pattern the developing embryo in the development of the axial skeleton and limbs. Hoxa13 and Hoxd10-Hoxd13 are expressed during specification of the hand/foot (autopod) (Favier and Dollé 1997). A variety of limb malformations including synpolydactyly and hand-foot-genital syndrome are known to be caused by specific mutations in HOXD13 and HOXA13, respectively (Muragaki et al. 1996; Mortlock and Innis 1997) In 2003, Wang identified 12 alleles at Hox4Ep-a microsatellite marker on *HoxD* gene; transmission of disequilibrium was found at the 12th allele, indicating that *HoxD* may be a potential gene for ICTEV (Wang et al. 2003). Wang and her colleagues, who identified the susceptibility of *HoxD* with ICTEV from the previous study group, further found SNP rs847154 located in 5′ flanking sequence of *HoxD12* gene and SNP rs13392701 located in exon 1 of HoxD13 to be associated with ICTEV (Wang et al. 2005). In another study investigating *HoxA* in ICTEV patients, the authors found seven alleles at D7S516 microsatellite and the presence of transmission disequilibrium in Chinese populations (Wang et al. 2008). Variants in *HoxA* and *HoxD* clusters and altered transmission in multiplex and simplex families were validated in a larger Western population with ICTEV in 2009 (Ester et al. 2009).

#### Collagen family genes

COL9A1 encodes one of the three alpha chains of Type IX collagen, which is a minor (5–20 %) collagen component of hyaline cartilage. Lack of Type IX collagen is associated with early onset of osteoarthritis, epiphyseal dysplasia, and intervertebral disc degeneration (Czarny-Ratajczak et al. 2001; Alizadeh et al. 2005; Boyd et al. 2008). Liu et al. studied COL9A1 which maps to chromosome 6q12-13 and found that 84 nuclear pedigrees had transmission disequilibrium in SNPs rs592121 and rs1135056, which are found in COL9A1(Liu et al. 2007). Expression of COL9A1 mRNA is significantly higher in patients with ICTEV than in healthy human subjects.

COL1A1 encodes the pro-alpha1 chains of Type I collagen, a fibril-forming collagen found in most connective tissues and abundant in bone, cornea, dermis, and tendon. Mutations in this gene are associated with osteogenesis imperfecta Types I–IV (Prockop et al. 1989; Takagi et al. 2015). Gene encoding collagen Type IV (COL1A1) was investigated in 2008 (Zhao et al. 2008). The results of this study show that expression of COL1A1 on mRNA levels is significantly higher in patients with ICTEV than in healthy patients. A −161(T → C) heterozygous mutation and a +274(C → G) homozygous mutation were also detected in the COL1A1 gene in patients with ICTEV, suggesting that COL1A1 mutations could cause ICTEV.

#### GLI3 gene

GLI3 encodes a protein which belongs to the C2H2-type zinc finger proteins subclass of the Gli family. The GLI3 protein localizes in the cytoplasm and activates patched Drosophila homolog (PTCH) gene expression. Mutations in the limb development related gene GLI3 have been associated with polydactyly (Volodarsky et al. 2014). SNP rs929387, located in exon 14 of the GLI3 gene, has transmission disequilibrium in 84 nuclear pedigrees, showing the association between the GLI3 gene and occurrence of ICTEV (Zha et al. 2006).

#### N-Acetylation genes

The NAT2 gene encodes an enzyme that functions to both activate and deactivate arylamine, hydrazine drugs and carcinogens. Polymorphisms in this gene are responsible for the N-acetylation polymorphism which in human populations segregates into rapid, intermediate, and slow acetylator phenotypes. Since smoking is one of the known environmental risk factors for ICTEV and NAT2 metabolizes tobacco byproducts, Hecht et al. (2007) examined the variants of the NAT2 gene in 56 ICTEV multiplex families, 57 trios with a positive family history, and 160 simplex individuals. They reported a slight decrease in the expected number of homozygotes for the NAT2 normal allele in the Hispanic simplex trios. Significantly slow NAT2 acetylator phenotype was detected among the ICTEV patients, suggesting slow acetylation may be a risk factor for ICTEV.

#### T-box family genes

A possible association between TBX3 and ICTEV has been reported. The TBX3 gene is a member of a phylogenetically conserved family of genes that share a common DNA-binding domain, the T-box. T-box genes encode transcription factors involved in the regulation of developmental processes. The TBX3 protein is a transcriptional repressor and mutations in this gene affect limb development. The third allele short tandem repeat (D12S378) in the region of chromosome 12q24, where the TBX3 gene is located, was proven to have transmission disequilibrium in ICTEV patients, suggesting that TBX3 is a susceptible gene for ICTEV (Ren et al. 2004). TBX4 shares a similar structure with TBX3. Expression studies in mice and chickens show that TBX4 is expressed in developing hindlimb but not forelimb buds, suggesting a potential role for this gene in regulating limb development and specification of limb identity (Dai et al. 2014; Menke et al. 2008). TBX4 microdeletions/microduplications have been found in individuals with clubfoot (Alvarado et al. 2010). However, another study which examined the possible correlation between hindfoot specific gene TBX4 and ICTEV concluded that (1) there was minimal evidence indicating an association between TBX4 and clubfoot; (2) no pathogenic sequence variants were identified in the two known TBX4 hindlimb enhancer elements (Lu et al. 2012). However, the PITX-TBX4 pathway was studied and further investigated by the common disease-rare gene supporters (Gurnett et al. 2008). The study concludes that PITX1 or its pathways may be etiologically responsible for the increased incidence of ICTEV.

#### Apoptotic pathway genes

Cysteine-dependent aspartate-directed proteases (Caspases) are a family of cysteine proteases that play essential roles in apoptosis (programmed cell death), necrosis, and inflammation (Alnemri et al. 1996). The association between caspase genes and ICTEV was first studied in 2005 (Heck et al. 2005). The authors reported that the major allele of a variant in the CASP10 gene, a gene in the apoptotic pathway, is associated with ICTEV in simplex white and Hispanic trios. Further examination on the mitochondrial apoptotic related genes was performed to investigate their association with ICTEV (Ester et al. 2007). One SNP in each of the apoptotic genes (Casp3, Casp8, Casp9, Casp10, Bid, Bcl-2, and Apaf1) provided evidence implying correlation with ICTEV, suggesting the potential role of genetic variation in apoptotic genes in development of ICTEV (Gahlmann and Kedes 1990).

#### Muscle contractile family genes

Troponin (Tn), a key protein complex in the regulation of striated muscle contraction, is composed of three subunits (Tn-I, Tn-T, and Tn-C). The Tn-I subunit inhibits actomyosin ATPase. The Tn-T subunit binds tropomyosin and Tn-C. The Tn-C subunit binds calcium and overcomes the inhibitory action of the troponin complex on actin filaments. TNNC2 encodes Tn-C subunit and plays a key role in initiating muscle contraction in fast-twitching muscle fibers (Mckillop and Geeves 1993). In 2011, Weymouth et al. studied the association of the muscle contractile genes with ICTEV and identified two muscle contractile genes (TNNC2 and TPM1) associated with ICTEV (Weymouth et al. 2011).

TPM1 is a member of the tropomyosin family of highly conserved, widely distributed actin-binding proteins involved in the contractile system of striated and smooth muscles and the cytoskeleton of non-muscle cells. Tropomyosin functions in association with the troponin complex to regulate the calcium-dependent interaction of actin and myosin during muscle contraction. The associations of multiple SNPs in the TPM1 gene with ICETV suggest a potential role of genes that encode contractile proteins of skeletal myofibers on the etiology of ICTEV (Shyy et al. 2010a, b).

#### Genome-wide association study

Besides the aforementioned candidate gene studies, a genome-wide association study was conducted in 396 isolated ICTEV patients and 1000 controls of European descent to identify novel genes for ICTEV (Zhang et al. 2014). The selected genetic variants from the genome-wide association study were further replicated with an independent cohort of 370 isolated ICTEV cases and 363 controls with the same ethnicity. The genome-wide association and replication study found an intergenic SNP on chromosome 12q24.31 between NCOR2 and ZNF664 that was significantly associated with ICTEV. However, Additional suggestive SNPs (Hox Genes, PITX1, TBX4, FOXN3, SORCS1 and MMP7/TMEM123) and identified pathways were not significant in the replication phase.

### Negative or controversial results

Shyy et al. (2009) studied two candidate genes (CAND2 and WNT7a) and tested the hypothesis that mutations in these genes would be associated with the phenotype of ICTEV. After sequencing ICTEV patients, they found a polymorphism in each gene. However, the association results indicated that CAND2 and WNT7a are not the major genes that cause ICETV. In a study exploring variation in MYH gene families, the authors sequenced the exons, splice sites, and predicted promoters of MYH genes in ICETV patients (Shyy et al. 2010a, b). They found many SNPs, but none proved to be significantly associated with the phenotype of ICTEV. Bonafé et al. conducted research on diastrophic dysplasia sulphate transporter gene (DTDST) to test whether R279 W mutations are responsible for occurrence of ICTEV (Bonafé et al. 2002). Alterations in the coding region were not identified in 10 probands with ICTEV and a positive family history. The authors concluded that the R279 W mutation is no more frequent in this population of ICTEV probands than in controls. Contrary to this finding, another author reported in 2009 that DTDST gene mutations were detected in 27 children with ICTEV, but in only two normal children in the Chinese population, indicating the possible role of DTDST in ICTEV (He et al. 2009).

### Other miscellaneous findings

In 2006, Sharp et al. found that children who carry the 677T variant of the MTHFR gene have a lower risk of ICTEV (Sharp et al. 2006). In 2009, Li and his colleagues compared the expression of CD-RAP (cartilage derived retinoic acid sensitive protein) in the abductor hallucis muscle from ICTEV and normal controls and found CD-RAP over-expressed in ICTEV patients, showing that CD-RAP might be a susceptibility gene of ICTEV (Li et al. 2009).

## Discussion

In this systematic review, positive associations between genetic variants and ICTEV were established in several studies. Certain genetic variants were found to have significant association with ICTEV. However, it must be noted that conflicting and negative results were also identified which do not necessarily undermine their contribution to the occurrence of ICTEV.

**Fig. 2:**
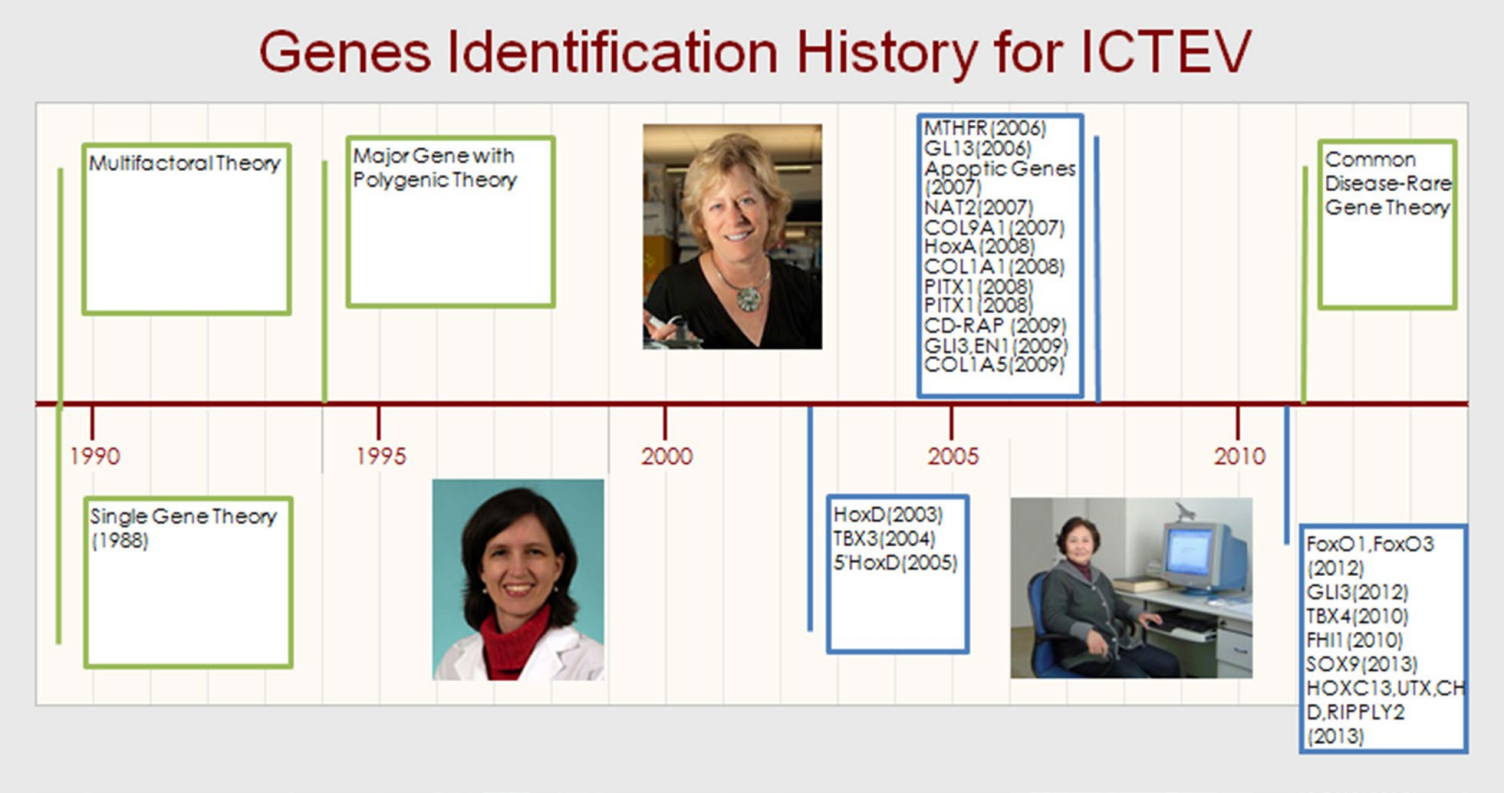
Genetic study history of ICTEV is shown. Different genes were identified in different years which are listed in *blue boxes*. The genetic theories to explain the etiology of ICTEV are marked in the *green boxes*. Several genes were identified and verified by Professor Jacqueline T. Hecht and her research group (under the timeline on the *left*); they identified and verified several genes in addition to caspase pathway changes in ICTEV patients. Dr. Christina A Gurnett (under on the timeline on the *right*) first used second generation sequencing methods to study ICTEV. Professor Chun-Lian Jin (above the timeline) pioneered clubfoot genetic study among Chinese patients

ICTEV’s genetic study history is described in Fig. 2. Simple major genes like X-linked genes, autosomal recessive genes and autosomal dominant genes used to be considered as possible genetic factors for ICTEV (Palmer 1964; Böök 1948; Wynne 1965). At the same time, another study concluded that multifactorial inheritance is significant in the etiology of ICTEV (Yamamoto 1979). Palmer et al. (1974) suggested simple major inheritance and multifactorial inheritance might be operating together to induce ICTEV. This theory was supported later by Wang et al. and Yang et al. who showed that a major gene component played a dominant role with additional minor contributions of multifactorial genes (Yang et al. 1987; Wang et al. 1988). In 1993, Rebbeck et al. (1993) rejected the non-Mendelian transmission pattern and concluded that the single Mendelian gene theory is adequate to explain the etiology of ICTEV. However, recent studies suggest that a polygenetic threshold model may explain its inheritance patterns. Contrary to the common disease-common variant hypothesis, one author introduced an alternative theory based on recent reports that rare genetic variants (with allele frequencies of <5 %) each confer a moderate risk with higher penetrance, which might be the genetic inheritance pattern of ICTEV. According to this theory, the PITX1-TBX4 transcriptional pathway directing early limb development is responsible for ICTEV (Dobbs and Christina 2012). However, further studies should be done to investigate other genes with low frequencies and how their variants affect ICTEV occurrence. Not only were gene mutations found in ICTEV, but chromosomal deletions and regulatory mutations were also reported.

## Conclusion

Several genes were identified, though none of them could solely explain the occurrence of ICTEV. Because the sample size of most association studies was small, most of the studies included are largely underpowered. In those included studies, rare coding variants were rarely investigated. Candidate genes were not replicated in larger ICTEV populations. These limitations should be addressed in future studies. In the future, genetic research on ICTEV could be focused on at least five aspects. First, high-throughput sequencing instead of GWA studies might be used to detect replicable candidate genes. The sample size should be calculated and candidate genes must be replicated in other studies. Second, epigenetic sequencing examining regulatory mechanisms for RNA could be studied. Third, novel genes like FOXN3 and SORCS1 identified by the GWAS may be further investigated. Studying genes and their interactions could reveal common pathways which are responsible for the occurrence of ICTEV. Their functions and interactions are worthy of clarification. Fourth, recent advances in chromosome conformation capture may show more structural changes on a chromosomal level (Imakaev et al. 2012). Three-dimensional variants may shed light on ICTEV etiology and treatment. Fifth, in clinical practice, some patients do not have any recurrence although they are not completely compliant with the brace treatment, whereas other patients have a recurrence even though they are strictly compliant with the brace treatment (Zhao et al. 2014). It is conceivable that certain genes being activated at certain times results in the relapse of ICTEV. Therefore, integration of genomic risk assessment alongside other clinical investigations may help personalize the treatment of ICTEV and improve the prognosis in the era of precision medicine (Castaneda et al. 2015).
